# Design and Validation of a Questionnaire to Assess the Leisure Time Physical Activity of Adult Women in Gipuzkoa

**DOI:** 10.3390/ijerph19095736

**Published:** 2022-05-08

**Authors:** Olaia Eizagirre-Sagastibeltza, Uxue Fernandez-Lasa, Javier Yanci, Estibaliz Romaratezabala, Ruth Cayero, Iñaki Iturrioz, Oidui Usabiaga

**Affiliations:** 1 Faculty of Education and Sport, University of the Basque Country (UPV/EHU), 01007 Vitoria-Gasteiz, Spain; oeizagirre003@ikasle.ehu.eus; 2 Society, Sport and Physical Activity (GIKAFIT) Research Group, Department of Physical Education and Sports, Faculty of Education and Sport, University of the Basque Country (UPV/EHU), Lasarteko Bidea 71, 01007 Vitoria-Gasteiz, Spain; javier.yanci@ehu.eus (J.Y.); estibaliz.romaratezabala@ehu.eus (E.R.); oidui.usabiaga@ehu.eus (O.U.); 3 Department of Physical Education and Sports, Faculty of Education and Sport, University of the Basque Country (UPV/EHU), 01007 Vitoria-Gasteiz, Spain; ruth.cayero@ehu.eus; 4 Diputación Foral de Gipuzkoa, Gipuzkoa Provincial Council, 20004 Donostia-San Sebastian, Spain; iiturrioz@gipuzkoa.eus

**Keywords:** women, LTPA, questionnaire, assessment, validation

## Abstract

Inactivity is higher among women than among men, and there are few specific questionnaires used to assess physical activity (PA) in women that are truly meaningful to them. This article tackles the design and validation process of an ad hoc multidimensional questionnaire to assess leisure time physical activity (LTPA) among adult women of Gipuzkoa. The questionnaire was completed by 3595 adult women (43.5 ± 12.1 years), 32% of which were inactive and 68% of which were active. Content validation, ecological validation, and internal consistency analysis results were satisfactory. The Gipuzkoa Women’s Physical Activity Questionnaire (GWPAQ) consists of four dimensions and 21 items. Barriers to PA were found related to intrapersonal, environmental, and socio-cultural aspects. The importance of family and spousal support in increasing PA levels was also observed. It is concluded that the GWPAQ is valid for obtaining evidence that can be used by public institutions to optimise women-specific PA promotion policies.

## 1. Introduction

Physical inactivity has been found to be among the main factors that increase disease and mortality rates, causing 6–10% of major non-communicable diseases worldwide [1,2]. In contrast, physical activity (PA) has been found to provide many health benefits [3]—both physical [4,5,6] and psychological [7,8,9,10]—and contribute to the prevention and reduction of risks associated with several diseases [11,12,13,14]. Due to the importance of PA, the World Health Organization (WHO) has established guidelines for PA practice, with variations for different age and population groups [15]. Physical inactivity is defined as failure to accumulate at least 150 min of moderate PA or 75 min of vigorous PA or the combination of both intensities per week, whereas being physically active is related to complying the guidelines established by the WHO [16].

Globally, it is estimated that 3 out of 10 people aged 15 or more do not meet the recommendation of at least 150 min of moderate PA per week [17]. Moreover, recent research continues to find that women engage in less PA than men [18,19]. On the one hand, stereotypes and gender roles are among the main factors contributing to this situation and that PA remains particularly male-oriented, which in turn may lead to greater drop out among women [20]. On the other hand, women are often directed towards taking responsibility for caregiving tasks and putting the needs of others before their own. This often results in little room for leisure [21,22] or low-quality leisure [23], which may affect their level of PA. Additionally, inactivity is heightened among working-age women due to the diversity of situations that occur at that life stage, such as entering the labour market, leaving parents’ home, motherhood, or caregiving [24,25,26,27]. Due to the plurality of factors and the interaction between them, women should be considered a heterogeneous group with diverse realities [28]. Accordingly, in addition to the feminist perspective on leisure and active life, socio-ecological perspectives could be of great use to address and understand this complexity, as they emphasise the relationships that people have with their physical and socio-cultural environment [29,30].

Public institutions generally aim to promote PA among the entire population [31,32]. However, despite many existing studies regarding the factors associated with inactivity and PA among different age groups [33,34,35] and especially among adult women [36,37], conducting a contextualised assessment before designing, developing, and implementing public policies to promote PA among women is still necessary and beneficial [38]. While considerable progress has been made in identifying women’s PA patterns, there is still a need to develop questionnaires that are meaningful to women, culturally relevant, short, and easily understood by people from diverse backgrounds [39]. In this regard, an ad hoc questionnaire may be an appropriate tool to learn about women’s perceptions of PA. Thus, public institutions responsible for promoting PA could have accurate initial information on the needs perceived by those involved and be able to accordingly propose effective actions. However, in addition to being adapted to the context of intervention, self-administered questionnaires that assess PA in adults should be easy to fill out for the target population and yield verified validity and measurement properties [40]. The aforementioned authors note the existence of numerous scales and questionnaires that assess PA in adults but stress that it is up to the researchers themselves to determine which questionnaire best suits their purposes. Consequently, available measurement tools may not always respond to the complexity of the study context, lack evidence of reliability or validity, or simply not be useful for the institution responsible for developing and implementing PA policies [40].

Considering the above, having questionnaires to determine both inactivity and PA levels that are validated and specific for distinct population groups seems to be essential. Therefore, the aims of the study were: (a) to design an ad hoc multidimensional tool to assess leisure time physical activity (LTPA) and leisure time sedentary behaviours of adult women (18–65 years) from Gipuzkoa (Spain); (b) to analyse the questionnaire’s content validity, ecological validity, and internal consistency; and (c) to describe LTPA and sedentary behaviours of adult women from Gipuzkoa.

## 2. Materials and Methods

### 2.1. Design

The present study was carried out in two phases. In the first phase, questionnaire design, content validation, and ecological validation were carried out by an expert panel. In the second phase, the questionnaire was administered to the target population to analyse item internal consistency and provide descriptive results. This work is part of a larger study called the Gipuzkoa Equitactive Study (GES).

### 2.2. Participants

In the first phase, six researchers prepared the questionnaire, and then, a panel of five experts in PA promotion for women was selected through purposive sampling to analyse content validity. Members of the expert panel were chosen both due to their extensive research careers and their training and academic experience in Physical Activity and Sport Sciences (between 15 and 30 years). For the ecological validity, three broadly experienced (between 27 and 35 years) sport management experts participated. Their expertise was in sport departments of public, regional, and municipal institutions in the specific context where the present study was carried out.

In the second phase, internal consistency of the items and data collection were analysed, for which the total population of adult women in Gipuzkoa (219,221 women) [40] was considered to estimate the minimum sample size needed (1006 women) (sampling error < 5%, 95% confidence interval, CI, α > 0.80). Furthermore, given population distribution, it was planned that the sample should be stratified by type of municipality of residence (rural: <5000 inhabitants; semi-urban: 5000–40,000; urban: >40,000) [41], participant age (18–24 years; 25–44; 45–65) [42], and PA level (>150 min/week of PA = active; <150 min/week of PA = inactive) [16]. The inclusion criteria were: (1) female, (2) aged between 18 and 65, and (3) residing in Gipuzkoa. A total of 3595 adult women (43.5 ± 12.1 years of age, sampling error = 1.5%, 95% CI, α > 0.95) completed the self-administered questionnaire. Of the total participants, 6.7% were aged 18–24, 45.7% were aged 25–44, and 47.7% were aged 45–65. Regarding the place of residence, 8.2% lived in rural municipalities, 50.9% in semi-urban municipalities, and 40.9% in urban municipalities. Inactive women represented 32% of the sample, whilst 68% were active. Lastly, 6.5% of the participants had no income, 3.6% had an income of less than EUR 499 per month, 7.8% between EUR 500 and 949, 20.1% between EUR 950 and 1499, 22.9% between EUR 1500 and 1999, 20.7% between EUR 2000 and 2499, 7.1% between EUR 2500 and 2999, 3.9% between EUR 3000 and 4999, and 6.6% did not answer this item.

All participants in this study answered the questionnaire voluntarily and were free to withdraw from the research at any time. The study was approved by the Ethics Committee for Human Research (CEISH, cod. M10-2020-296) of the University of the Basque Country (UPV/EHU) and followed the guidelines established in the Declaration of Helsinki [43].

### 2.3. Procedure

The present study was requested by the Gipuzkoa Provincial Council, which commissioned the research group to carry out an assessment of the PA habits of adult Gipuzkoan women in order to optimise PA promotion policies. In an effort to conduct an evidence-based intervention, in line with Heath et al. [44], and with the proven effectiveness of a participatory and co-creative process [45,46,47], a working group was established with two researchers from the research group and three sport managers from the Gipuzkoa Provincial Council. The group identified several relevant dimensions, such as PA level, motives for participating in PA, and barriers to PA. Based on an in-depth analysis of the existing literature, the first draft of the ad hoc multidimensional questionnaire was drawn up. The content of this first version was validated by a five-person expert panel. A similar approach was taken for ecological validity, with the three sport managers suggesting several improvements. Once the experts’ contributions had been reviewed, and the final version was ready, the questionnaire was sent out by email to the 21,000 women who were registered in the databases of the requesting institution. These women were also invited to forward the message to as many women as they considered appropriate. Three days later, another message was sent to several women’s associations contacts in Gipuzkoa via an instant messaging app.

### 2.4. Measuring Instrument

Gipuzkoa Women’s Physical Activity Questionnaire (GWPAQ): the multidimensional questionnaire in this study was developed under the postulates of the socio-ecological [48,49] and feminist perspectives of leisure and active life [28,50,51]. The GWPAQ (File S1) includes four dimensions (level of LTPA, sedentary habits, LTPA habits, and family-life balance for LTPA participation) and 21 items. The third dimension, regarding LTPA habits, is divided into two sub-dimensions: one focused on the characteristics and motives for LTPA participation among active women and the other aimed at identifying the barriers encountered by inactive women to participate in LTPA and their intention to change this situation. Most items had only one answer to choose from. However, multiple choices were admitted for the items regarding the motives or barriers to LTPA participation or the collaboration of participants’ family, friends, or acquaintances.

### 2.5. Statistical Analysis

Results are presented as mean ± standard deviation (SD), frequencies, and percentages. Cronbach’s alpha statistic was used to describe the internal consistency of the questionnaire. For each questionnaire item, an independent samples test or Pearson’s chi-square test was used to analyse differences between the active and inactive groups. Where possible, effect size (ES) of the differences was calculated [52]. ES was classified as trivial (<0.2), low (0.2–0.5), moderate (0.5–0.8), and high (>0.8) [52]. The analysis was conducted with the Statistical Package for Social Sciences (SPSS Inc., version 26.0, Chicago, IL, USA). Statistical significance was set at *p* < 0.05.

## 3. Results

### 3.1. Questionnaire Content Validity

Listed below are the most relevant qualitative assessments made by the expert panel on the initial dimensions and items considered for the final GWPAQ design. Regarding the content, all experts answered that both a dimension focused on family-life balance for LTPA participation and another focused on sedentary habits were lacking. Likewise, regarding the adequacy of items belonging to the same dimension, they stressed the need to add other options in the questions on barriers to PA. They also suggested dividing the ages of children in the items related to motherhood into shorter brackets. Concerning item clarity, experts found it necessary to reformulate some items to increase their understandability and to add or modify some examples used in the questions on intensity level or type of LTPA. Correction of errors related to terms such as physical exercise, sport and PA, free time, and leisure time were also requested. Literature review wise, experts suggested including the contributions of several studies aimed at understanding the collaboration (or non-collaboration) of people within the close environment of women (partners, family members, and friends).

### 3.2. Questionnaire Ecological Validity

Once the initial questionnaire had been optimised based on the experts’ contributions, the professional sport managers team of the Provincial Council of Gipuzkoa also suggested modifications, which were included in the questionnaire. They mainly requested a greater specification of PA levels in order to classify inactive and active women more clearly. They also highlighted that adding a question regarding participation in women-only PA programmes to the habits dimension might be relevant. Specifically, they suggested adding examples of programmes implemented in the context of the study. Along the same lines, they asked to add another question about inactive women’s knowledge about the Mugiment project (https://mugiment.euskadi.eus/homepage/, accessed on 24 January 2021). Lastly, they suggested adding more examples of PA types or substituting them with other locally well-known ones. Along the same lines, they found it necessary to add other barriers to PA that they had detected in their professional development as sport managers.

### 3.3. Questionnaire Internal Consistency

Regarding between-items internal consistency of the different dimensions, Cronbach’s alpha values were: 0.634 for dimension D1 (level of PA), 0.226 for D2 (sedentary habits), 0.940 for D3 active (LTPA habits of active women), 0.813 for D3 inactive (LTPA habits of inactive women), and 0.298 for D4 (family-life balance for LTPA participation).

### 3.4. Questionnaire Results

#### 3.4.1. Dimension 1. Level of LTPA

**Figure 1:**
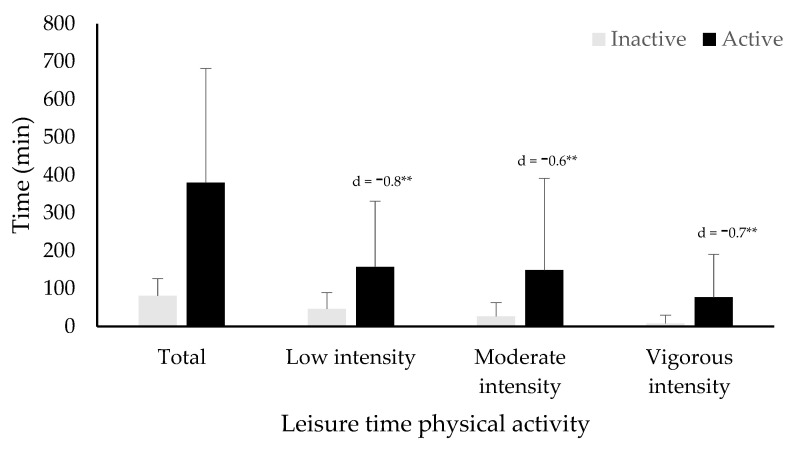
Results of weekly LTPA minutes for both inactive (<150 min per week) and active (>150 min per week) women. ** *p* < 0.001, significant differences between the inactive and active groups.

Results show that 32% of the respondents (inactive group) currently perform less than 150 min of LTPA per week, while the remaining 68% (active group) performed 150 min or more per week. Mean weekly LTPA added up to a total of 281.6 ± 285.8 min, with 120.9 ± 153.6 min of low intensity PA, 108.7 ± 207.9 min of moderate intensity, and 53.7 ± 98.8 min of vigorous intensity PA. Figure 1 shows the results of the weekly LTPA minutes for both inactive and active women.

#### 3.4.2. Dimension 2. Sedentary Habits

Table 1 shows the results regarding sedentary habits both for the total sample and for the two groups (inactive vs. active). The response distribution of both groups was significantly different (*p* < 0.01).

#### 3.4.3. Dimension 3. LTPA Habits

**Figure 2:**
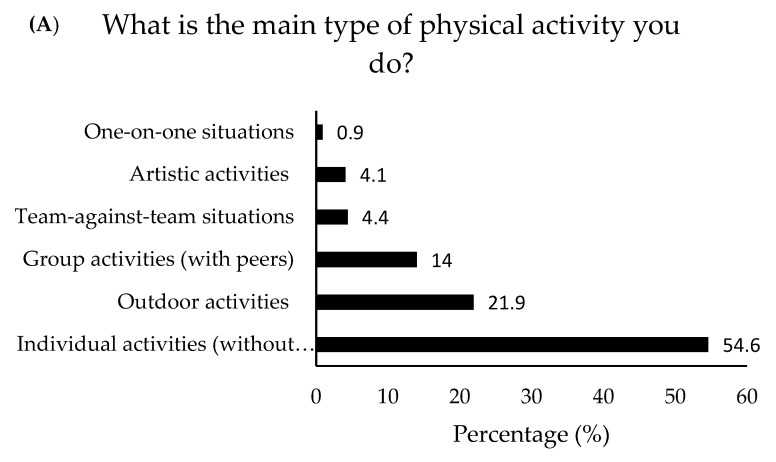

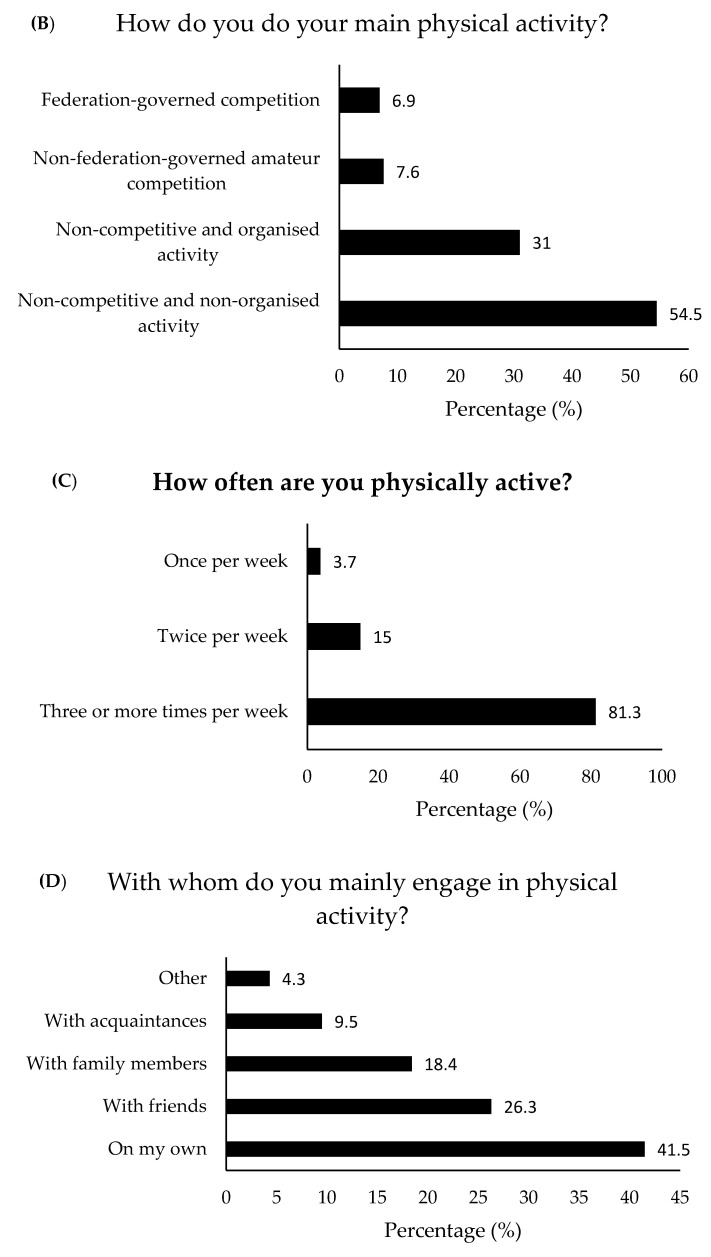
Active group participant responses regarding the type and characteristics of LTPA.

*Motives and types of LTPA* (*active group*): Table 2 shows the results concerning the motives for being physically active by active women (>150 min/week, 68% of total, *n* = 2458 women). Figure 2A–D shows the results concerning the type and characteristics of LTPA.

*Barriers to LTPA participation and intention to change* (*inactive group*): The results concerning the main barriers to being physically inactive in LTPA among inactive women (<150 min/week, 32% of the total, *n* = 1137 women) are shown in Table 3.

Within the inactive group, 11.7% perceived their health as very good, 63.6% as good, 20.8% as fair, 3.1% as bad, 0.0% as very bad, and 0.8% did not answer the question.

The answers to question “Before you stopped, for how long were you physically active?” showed that 10.2% of the participants had never performed PA or sport before, 7.2% had done so for less than a year, 11.4% had for 1 or 2 years, 13.3% for 3–4 years, 8.1% for 5–6 years, 35.3% for over 6 years, and 14.4% did not answer the question. Responses of the inactive group participants regarding the reasons for giving up LTPA are shown in Table 4.

On the other hand, 36.1% of the inactive women answered that they were sure they would restart LTPA, 40.6% that it was likely, 16.2% that they did not know, 3.5% that they probably would not, 0.8% that they were sure they would not, and 2.8% did not answer the question. Results regarding the motives why inactive participants might restart LTPA are shown in Table 5.

A total of 81.1% of the inactive women stated that they were aware of either the Mugiment project, which unites PA-promoting and sedentary lifestyle-reducing activities and is aimed at achieving an active Basque society, or programmes associated with it. Only 17.3% were not aware of it, and 1.6% did not answer this question.

*Participation in women-only PA programmes* (*inactive and active groups*): Out of all the participants, 76.6% had never participated in any specific women-only PA programme or course versus 23.4% who had. However, among the inactive women (32% of the total), 83.1% had not participated in such events, while 16.9% had. By contrast, among the active women (68% of the total), 73.6% had not participated in any specific women-only PA programme or course, while 26.4% had.

#### 3.4.4. Dimension 4. Family-Life Balance for LTPA Participation

In the item on motherhood, 42.6% of the participants stated that they were not mothers or were not responsible for any minors, while the remaining 57.4% did have those responsibilities. Among the inactive group, only 32.5% had no child-related responsibilities versus 67.5% that did. However, among the active women, 64.2% were not mothers or responsible for any minors, and only 35.8% were. Significant differences (*p* < 0.001) were found in response distribution between the inactive and active groups.

Regarding the care of dependent persons (relatives, dependent elderly, disabled, or ill), 88.6% did not engage in these tasks, while 11.4% did. Inactive women were significantly more involved in caregiving than active ones (14.3% vs. 10.1%, *p* < 0.01).

Responses from items referring to support received from partners, family, or friends to engage in LTPA are shown in Table 6.

## 4. Discussion

The main purpose of this study was to develop a valid multidimensional questionnaire to measure and describe LTPA participation and sedentary behaviours of adult women in Gipuzkoa as a basis for the implementation of specific policies by the Provincial Council of Gipuzkoa to promote PA. Though self-administered questionnaires designed to measure PA level and assess sedentary behaviours among adults [40,53,54,55,56,57], according to Ainsworth [39], the design and validation of ad hoc questionnaires aimed exclusively at women is still needed. These questionnaires should address the gender-specific barriers to LTPA participation perceived by women and consider their heterogeneous and diverse nature [28]. Likewise, socio-ecological perspectives [30] might help understand the reasons behind these women’s physical inactivity based on their circumstances and provide relevant information about their physical and socio-cultural environment. Therefore, we tried to ensure that the data extracted from the GWPAQ represent the complex reality of adult women of Gipuzkoa. With this approach, it would be possible to identify and understand the factors leading women to inactivity and provide public institutions with evidence aimed at designing, developing, and implementing suitable strategies for PA promotion.

### 4.1. Content Validity, Ecological Validity, and Internal Consistency

Questionnaire validation is necessary before application and is usually carried out by researchers [55,57,58]. One of the most common methodologies is content validation [59]. In the present study, six experts designed the questionnaire, which was later assessed by an expert panel in women’s PA promotion. Their contribution led to modifications of content, adequacy, and clarity in several dimensions and items. Furthermore, given the questionnaire was aimed at a specific population—the adult women of Gipuzkoa—its ecological validity was assessed by three sport management experts, as Sabariego et al. [60] did. Their main contributions were related to content contextualisation and barriers to LTPA in terms of usefulness, suitability, and coherence. Lastly, the internal consistency of all items was evaluated, with acceptable results. Though results for dimensions D1, D3 active, and D3 inactive were good or very good, results for dimensions D2 and D4 were poor. In the case of D2, a two-item dimension regarding sedentary habits asking about the total level of sedentariness in one question and about the level of sedentariness during leisure time in the other may have conditioned internal consistency. Similarly, the questions in D4 (family-life balance for LTPA participation) are addressed to two different aspects (partner and family), which may have led to lower internal consistency.

### 4.2. Dimension 1. Level of LTPA

PA has numerous health benefits and can contribute to prevent several diseases [61,62,63]. Among women, it has been found to aid in the prevention of cardiovascular disease, diabetes, colon and breast cancer, and fatigue reduction, among others [64,65,66]. In the present study, active women not only performed more LTPA than inactive women overall, but they also performed more LTPA regardless of the intensity. Consistent with general PA levels among women [17], almost one-third of the participants did not reach the minimum recommendations for total PA, and LTPA participation of inactive women at low, moderate, and vigorous intensities was very low. Although more than two-thirds of the participants considered themselves active, average moderate-intensity PA practice was less than 150 min per week. In addition, the average time dedicated to vigorous-intensity PA was only slightly more than recommended (between 75 and 150 min) among active women. Due to its countless benefits, it seems necessary to encourage PA among all women [67] by promoting policies that support active life through the collaboration of different institutions and agents [68].

### 4.3. Dimension 2. Sedentary Habits

Like physical inactivity, sedentary habits increase the risk of mortality, especially when adults sit for more than seven hours per day. Both are factors in the development of diseases such as colon and breast cancer, diabetes, and coronary heart disease [69,70]. Almost half of the participants in both the active and inactive groups sat for over six hours per day on average, which is consistent with the results obtained by Strain et al. [71]. In terms of sedentary behaviours during leisure time, most participants in both groups spent less than two hours per day watching TV, using the computer, reading, or doing similar activities. However, Prince et al. [72] found that women aged 18–65 spent approximately three hours per day doing these types of activities. Policies aimed at reducing sedentary behaviour and increasing LTPA are needed, as they contribute to the prevention of cardiovascular disease and premature mortality [73], among other benefits.

### 4.4. Dimension 3. Active Women and Motives for and Types of LTPA

Understanding both the type of LTPA and the reasons that motivate it is useful to adapt public PA promotion policies and programmes to the needs of the population. Active women in this study engaged in LTPA to be fit, to prevent or improve health issues, to improve mood, to lose or maintain weight, or to simply enjoy exercise for entertainment. This partially matches the results obtained by Larsen et al. [74], where improving fitness was found to be the most important reason, followed by enjoyment and improving sports performance. Furthermore, several studies have found that women tend to engage in PA motivated by aspects related to physical appearance, physical and psychological well-being, and self-esteem [75]. Engaging in PA mainly for appearance and body weight control issues, however, may lead to body image dissatisfaction, unhealthy eating habits, or low self-esteem [76]. Therefore, although PA may help to improve body image in some cases, it is important to focus on the physical and psychological health benefits and the pleasure it brings [77].

The type of LTPA that was mostly performed by the active women in this study were non-competitive individual and/or outdoor activities. Frequency was mostly three or more times per week either alone or with family and friends. Although team sports offer an opportunity for socialisation and an enjoyable environment [78], many women do not find competitive PA attractive and prefer to focus on the social, physical, and psychological benefits [79,80]. Many programmes have been conducted to promote PA among women by using a variety of activities, with positive results [81,82,83,84]. Therefore, although activity type may influence participation, it is important to also consider factors such as intensity, frequency, or the physical and social environment where it takes place.

### 4.5. Dimension 3. Inactive Women and Barriers to LTPA and Intention to Change

A better understanding of the motives for why inactive people struggles to comply with PA recommendations could help develop strategies and resources to overcome these barriers. In this study, lack of time was the main motive why inactive women quit or did not practice enough LTPA. Laziness, tiredness due to work or studies, excessive workload, prioritising time spent with the family, and family responsibilities were other reasons for not being physically active. According to some studies, lack of time limits LTPA participation and is also closely linked to work requirements, family and home-related care work, and resulting tiredness [85,86]. Gender differences in leisure quality are smaller in countries with more egalitarian role expectations, institutionalised egalitarian norms regarding caregiving, and greater political power for women [23]. In turn, the greater the equality between women and men, the more LTPA women engage in [87]. Therefore, it may be necessary to establish policies aimed at reducing gender inequalities in this area, too, since LTPA participation is affected by a combination of factors.

Furthermore, despite that over a quarter of inactive women stated that they did not engage in any activity or had done it for less than two years before giving it up, most of them were interested in and willing to restart LTPA. Additionally, and according to Glanz et al. [88] and Hayotte et al. [89], it is important to identify the life-stage each person is at in regard to their intention to engage in LTPA so as to implement individually tailored strategies.

### 4.6. Dimension 3. Participation in Women-Only Programmes (Inactive and Active Groups)

Most of the participants (both active and inactive) had not participated in any women-only PA programmes. This type of schemes can be particularly effective, as they not only yield physical and psychological benefits but provide a safe and supportive space for women to engage in an activity with each other [90,91]. However, other studies have found that despite the implementation of PA promotion campaigns at the national level, difficulties in adapting to each environment may hinder implementation at the local level [92]. Developing programmes based on participants’ needs and their environment favours their implementation and success [38].

### 4.7. Dimension 4. Family-Life Balance for LTPA Participation

Many women often take on caregiving responsibilities and lack time for LTPA [82,93]. Particularly, women with children under 6 years of age are more likely to be inactive due to the time-consuming nature of childcare tasks, resulting in little time for personal leisure activities [94,95]. In line with the aforementioned studies, more than two-thirds of the inactive women in the current study were mothers or were in charge of a child. Having the support of partners, family, or friends can help with work-life balance and being able to devote time to LTPA [96,97]. Women in the active group reported higher family and spousal support to participate in LTPA, which indicates that having a support network might help to increase the level of LTPA and maintain it over time.

However, other studies [98,99,100] have found that some mothers who left their children in someone else’s care felt guilty and even selfish. At the same time, however, they felt that LTPA participation was positive both for themselves and for their family, as not only did they set an example, but they transferred the happiness and enjoyment generated through LTPA to the rest of the family [98,99,100]. For this reason, LTPA-encouraging policies should focus on improving work-family balance, encouraging partners and family/friends to support women in LTPA practice, and aiming to reduce any negative care-related feelings of guilt and neglect.

## 5. Conclusions

Based on the results of this study, the GWPAQ has proven a valid instrument for the assessment of LTPA among adult women of Gipuzkoa. This questionnaire somewhat meets the need to create ad hoc tools aimed exclusively at the study of women’s LTPA. On the other hand, it appears that one-third of the women in Gipuzkoa are inactive, and the difference in the time dedicated to LTPA between inactive and active women is statistically significant. Likewise, a large proportion of active and inactive women spend more than seven hours per day sitting down. This illustrates the need to increase time devoted to LTPA not only for the physical, psychological, and social benefits it entails but also to minimise the harmful effects of sedentary habits. Additionally, several barriers to LTPA were identified. Therefore, to make LTPA promotion effective, it is important to address the many needs and interests of women and to factor in their reality and physical and socio-cultural environments. Lastly, the results obtained in this study regarding work-life balance seem to show that family and partner support may be key when it comes to LTPA, and it is therefore advisable to develop policies that favour the management of women’s personal, family, and working lives.

## Figures and Tables

**Table 1:** Sedentary habits dimension results (%): total, inactive (<150 min/week of PA), and active (>150 min/week of PA).

	**On a typical day, how many minutes in total do you spend sitting? For example, studying, working, in meetings, commuting to school or work (car, motorbike, train, bus, or similar).**			**On a typical day, how many minutes of your leisure time do you spend watching TV, in front of the computer, reading, or doing similar activities?**		
**Time**	**Total**	**Inactive**	**Active #**	**Total**	**Inactive**	Active **
Less than 60 min (1 h)	5.2	2.1	3.1	28.3	10.3	18.0
Between 61–120 min (1–2 h)	9.4	3.1	6.3	38.9	10.4	28.5
Between 121–180 min (2–3 h)	8.8	2.3	6.5	17.9	4.8	13.0
Between 181–240 min (3–4 h)	8.0	2.3	5.7	6.5	2.3	4.3
Between 241–300 min (4–5 h)	10.5	2.9	7.6	3.1	1.4	1.7
Between 301–360 min (5–6 h)	13.1	4.0	9.1	1.7	0.6	1.0
More than 360 min (more than 6 h)	45.0	14.9	30.1	3.7	1.8	1.9

# Significant differences in response distribution, Pearson’s chi-square test (*p* = 0.009). ** Significant differences in response distribution, Pearson’s chi-square test (*p* < 0.001).

**Table 2:** Active group participant responses regarding the motives for being physically active in LTPA (*n* = 2458 women, total responses = 13,686).

Motives for Being Physically Active	Frequency	%
Be fit	2044	14.9
Exercise is good entertainment for me	1509	11.0
Avoid or manage health conditions	1424	10.4
Improve mood	1404	10.3
Lose or maintain weight	1268	9.3
Improve my body’s appearance	1143	8.4
Physical activity lets me have contact with friends and persons I enjoy.	1011	7.4
Improve my self-esteem	986	7.2
Physical activity gives me a sense of personal accomplishment	952	7.0
Improve athletic performance	589	4.3
Participate in social activities	453	3.3
Sharing activities with other women	359	2.6
Play with children/grandchildren/nephew/niece	257	1.9
As a consequence of the confinement during the pandemic	130	0.9
Other	100	0.7
Exercising increases my acceptance by others	35	0.3
Gain weight	22	0.2

**Table 3:** Responses of participants in the inactive group regarding barriers to LTPA (*n* = 1137 women, total responses = 3815).

Barriers for Not Being Physically Active	Frequency	%
Lack of time	777	20.4
Laziness	427	11.2
Fatigue due to work or studies	426	11.2
Overwork	347	9.1
Physical activity takes too much time from family relationships and family responsibilities	293	7.7
The weather puts me off	206	5.4
Prefer to do other things	190	5.0
I have nobody to go with	173	4.5
I do not enjoy physical activity	145	3.8
Lack of confidence	101	2.6
Ill health, injury, or disability	97	2.5
Other	93	2.4
I am too embarrassed to exercise	90	2.4
I feel too fat/overweight	89	2.3
Lack of money	88	2.3
Lack of adequate facilities in my area	78	2.0
Sense of insecurity (darkness, unknown areas)	69	1.8
Feeling that my physical appearance is worse than that of others	63	1.7
I think I look ridiculous in exercise clothes	19	0.5
I do not like doing exercise	19	0.5
I am not comfortable with people exercising with me	10	0.3
Do not know/No answer	7	0.2
Lack of transport	4	0.1
Lack of suitable monitors/trainers	4	0.1

**Table 4:** Inactive group participant responses concerning the motives for giving up PA (total *n* of responses = 1964).

Which Are the Most Important Motives for Giving Up Physical Activity?	Frequency	%
Lack of time	755	38.4
Laziness	755	38.4
Physical activity takes too much time from family relationships and family responsibilities	258	13.1
Prefer to do other things	169	8.6
Overwork	169	8.6
I have nobody to go with	133	6.8
I do not like doing exercise	133	6.8
Ill health, injury, or disability	119	6.1
Feeling that my physical appearance is worse than that of others	119	6.1
I do not enjoy physical activity	115	5.9
Lack of adequate facilities in my area	115	5.9
The weather puts me off	112	5.7
Fatigue due to work or studies	112	5.7
Lack of money	95	4.8
Lack of suitable monitors/trainers	95	4.8
Lack of confidence	72	3.7
Other	72	3.7
I feel too fat/overweight	49	2.5
I am not comfortable with people exercising with me	49	2.5
I am too embarrassed to exercise	42	2.1
Sense of insecurity (darkness, unknown areas)	31	1.6
I think I look ridiculous in exercise clothes	9	0.5
Lack of transport	5	0.3
Do not know/No answer	5	0.3

**Table 5:** Inactive group participant responses regarding the motives for why they might restart LTPA (total responses (*n*) = 4669).

Choose the Most Important Motives for Restarting LTPA	Frequency	%
Be fit	797	17.1
Improve mood	534	11.4
Avoid or manage health conditions	513	11.0
Improve my body’s appearance	470	10.1
Lose or maintain weight	469	10.0
Other	469	10.0
Improve my self-esteem	396	8.5
Physical activity gives me a sense of personal accomplishment	364	7.8
Exercise is good entertainment for me	348	7.5
Physical activity lets me have contact with friends and persons I enjoy	160	3.4
Play with children/grandchildren/nephew/niece	135	2.9
Improve athletic performance	134	2.9
Participate in social activities	130	2.8
As a consequence of the confinement during the pandemic	96	2.1
Sharing activities with other women	94	2.0
Exercising increases my acceptance by others	22	0.5
Gain weight	7	0.1
Do not know/No answer	7	0.1

**Table 6:** Responses (%) of total, inactive, and active group participants regarding support from partners, family members, or friends to engage in LTPA.

**Does Your Partner Help You to Do More PA in Your Leisure Time?**	**Total**	**Inactive**	Active **
I don’t have a partner	21.8	20.4	22.4
Has encouraged me to participate in physical activity	36.6	40.6	34.7
Has participated in physical activity with me	21.9	10.8	27.1
Has helped me to plan to take part in some physical activity	2.1	2.3	2.0
Has taken care of some of my duties so that I can do more physical activity	3.9	3.4	4.1
Has taken responsibility for childcare so that I could be more active	3.3	4.6	2.7
Has not offered me any help to be able to participate in physical activity	4.8	9.4	2.6
Has made it difficult for me to participate in any physical activity	0.1	0.3	0.1
Don’t know/No answer	5.6	8.2	4.4
**Do Your Family Members and/or Friends Help You to Be More Physically Active in Your Leisure Time?**	**Total**	**Inactive**	Active **
Have encouraged me to participate in some physical activity	38.9	39.9	38.4
Have participated in physical activity with me	25.7	14.6	30.8
Have helped me plan to participate in physical activity	1.5	1.0	1.7
Have taken care of some of my duties so that I could be more physically active	2.6	2.6	2.7
Have taken responsibility for childcare so that I could be more active	2.9	3.4	2.6
Have not offered me any help to participate in physical activity	12.5	18.6	9.6
Have made it difficult for me to participate in any physical activity	0.2	0.1	0.2
Don’t know/No answer	15.8	19.9	13.9

** Significant differences in response distribution, Pearson’s chi-square test (*p* < 0.001).

## Supplementary Materials

The following supporting information can be downloaded at: https://www.mdpi.com/article/10.3390/ijerph19095736/s1, File S1: GWPAQ questionnaire.
