# Supplementary material for: Design and Validation of a Questionnaire to Assess the Leisure Time Physical Activity of Adult Women in Gipuzkoa
Source: Int J Environ Res Public Health. 2022 May 8;19(9):5736. doi: 10.3390/ijerph19095736 (PMC9103327; doi:10.3390/ijerph19095736)
Supplement: Supplementary file 1 [file ijerph-19-05736-s001.zip › ijerph-1678453-supplementary.pdf]

File S1
Gipuzkoa Women's Physical Activity Questionnaire (GWPAQ)

The aim of this questionnaire is to understand the habits, motives and barriers regarding leisure time physical activity (LTPA) among adult women (aged 18 to 65) of Gipuzkoa. Answer the following questions:

DIMENSION 1. LEVEL OF LEISURE TIME PHYSICAL ACTIVITY

Q1. Currently, how much time do you spend doing physical activity in your leisure time during a typical week? *For example: walking, hiking, cycling, dancing, playing Basque pelota, yoga, playing football, swimming, or other physical activity. Please choose one of the following answers.*

- ☐ Less than 150 minutes per week
- ☐ 150 minutes per week or more

Q2. Exactly how many minutes do you spend doing physical activity in a typical week. *If you are not physically active at any level of intensity, enter 0 minutes.*

Total time of leisure time physical activity in a week: _____ minutes.

Enter how many minutes of the total are low intensity, how many are moderate intensity and how many are vigorous intensity.

Intensity Level	Minutes
**Low**<br>*When you demand minimum effort from your body and talking is easy during the activity. For example: leisurely walking or cycling, yoga, stretching, or other similar intensity activities.*	
**Moderate**<br>*When you push your body a little further, breathing becomes a bit laboured and talking is not easy anymore. For example: walking, cycling, fast swimming, brisk Nordic walking, dancing, running at an easy pace, a leisurely walk in the countryside, or other similar intensity activities.*	
**Vigorous**<br>*When you really exert yourself, your heart rate rockets, and talking becomes very difficult or impossible. For example: a hard aerobic or spinning session, very fast uphill walking, competing in sports such as football, basketball, handball, judo, Basque pelota or similar, surfing, cycling on a hilly course, running fast, a hard CrossFit session, or other similar intensity activities.*	

DIMENSION 2. SEDENTARY HABITS

Q3. On a typical day, how many minutes in total do you spend sitting? *For example, studying, working, in meetings, commuting to school or work (car, motorbike, train, bus or similar).*

- ☐ Less than 60 minutes (1 hour)
- ☐ Between 61-120 minutes (1-2 hours)
- ☐ Between 121-180 minutes (2-3 hours)
- ☐ Between 181-240 minutes (3-4 hours)
- ☐ Between 241-300 minutes (4-5 hours)
- ☐ Between 301-360 minutes (5-6 hours)
- ☐ More than 360 minutes (More than 6 hours)

Q4. On a typical day, how many minutes of your leisure time do you spend watching TV, in front of the computer, reading or doing similar activities?

- Less than 60 minutes (1 hour)
- Between 61-120 minutes (1-2 hours)
- Between 121-180 minutes (2-3 hours)
- Between 181-240 minutes (3-4 hours)
- Between 241-300 minutes (4-5 hours)
- Between 301-360 minutes (5-6 hours)
- More than 360 minutes (More than 6 hours)

DIMENSION 3. LEISURE TIME PHYSICAL ACTIVITY HABITS

If you do any physical activity, please answer questions Q5-Q9 and Q17. If you do not do any physical activity, continue from question Q10:

Q5. From the following options, which are the most important motives for being physically active?
Choose as many answers as you want.

- Be fit
- Exercise is good entertainment for me
- Avoid or manage health conditions
- Improve mood
- Lose or maintain weight
- Improve my body's appearance
- Physical activity lets me have contact with friends and persons I enjoy.
- Improve my self-esteem
- Physical activity gives me a sense of personal accomplishment
- Improve athletic performance
- Participate in social activities
- Sharing activities with other women
- Play with children/grandchildren/nephew/niece
- As a consequence of the confinement during the pandemic
- Exercising increases my acceptance by others
- Gain weight
- Other

Q6. What is the main type of physical activity you do? *Choose only one answer.*

- **Artistic activities.** For example: dance (Basque dances, lindy hop, funky, samba, tango, zumba, aerobics, etc.), acrobatic physical activities, etc.
- **Outdoor activities.** For example: hiking, mountain biking, surfing, canoeing, skiing, climbing, diving, rowing, and similar activities.
- **Individual activities (without partners).** For example: walking, running, rollerblading, cycling, swimming, strength training at the gym, Pilates, yoga, aqua gym, spinning, Basque rural sports (alone), etc.
- **Group activities (with peers).** For example: walking in a group, cycling in a cycle touring group, Nordic walking groups, etc.
- **One-on-one situations.** For example: tennis, Basque pelota, judo, karate, etc.
- **Team-against-team situations.** For example: football, handball, basketball, volleyball, rugby, doubles tennis, Basque pelota in pairs, etc.
- **Other types of activities**. Which? ....

Q7. How do you do your main physical activity? *Please choose only one answer.*

- **Non-competitive and non-organised activity.** For example, renting a court and playing a game or walking in the countryside.
- **Non-competitive and organised activity.** For example, signing up and taking part in courses at sports centres or dance academies, or taking part in outings organised by mountain clubs.
- **Amateur non-federation governed competition.** Training sessions are focused on specific events. For example, amateur races, cyclo sportives, Behobia-San Sebastián, local championships, etc.
- **Federation governed competition.** Participating in a competition organised by a sports federation on behalf of a club.

Q8. How often are you physically active? *Please choose only one answer.*

- Once per week
- Twice per week
- Three or more times per week

Q9. With whom do you mainly engage in physical activity? *Please choose only one answer.*

- On my own
- With family members
- With friends
- With acquaintances
- Other, who?...

Q10. Which are the most important barriers for not being physically active? *Choose as many answers as you want.*

- Lack of time
- Laziness
- Fatigue due to work or studies
- Overwork
- Physical activity takes too much time from family relationships and family responsibilities
- The weather puts me off
- Prefer to do other things
- I have nobody to go with
- I do not enjoy physical activity
- Lack of confidence
- Ill health, injury or disability
- I am too embarrassed to exercise
- I feel too fat/overweight
- Lack of money
- Lack of adequate facilities in my area
- Sense of insecurity (darkness, unknown areas)
- Feeling that my physical appearance is worse than that of others
- I think I look ridiculous in exercise clothes
- I do not like doing exercise
- I am not comfortable with people exercising with me
- Lack of transport
- Lack of suitable monitors/trainers
- Other

- Do not know / No answer

Q11. What is your health status like? *Choose the answer that best reflects your situation. Please choose only one answer.*

- Very good
- Good
- Fair
- Bad
- Very bad
- Don't know / No answer

Q12. Before you stopped, for how long were you physically active? *Choose the answer that best reflects your situation. Please choose only one answer.*

- I have never done any physical activity or sport
- Less than one year
- Between 1 and 2 years
- 3-4 years
- 5-6 years
- More than 6 years

Q13. Select the main barriers that have influenced you to not practice or quit physical activity. *Choose the answers that best reflect your situation. Choose as many answers as you want.*

- Lack of time
- Laziness
- Fatigue due to work or studies
- Overwork
- Physical activity takes too much time from family relationships and family responsibilities
- The weather puts me off
- Prefer to do other things
- I have nobody to go with
- I do not enjoy physical activity
- Lack of confidence
- Ill health, injury or disability
- I am too embarrassed to exercise
- I feel too fat/overweight
- Lack of money
- Lack of adequate facilities in my area
- Sense of insecurity (darkness, unknown areas)
- Feeling that my physical appearance is worse than that of others
- I think I look ridiculous in exercise clothes
- I do not like doing exercise
- I am not comfortable with people exercising with me
- Lack of transport
- Lack of suitable instructors/trainers
- Other
- Do not know / No answer

Q14. Do you think you will take up physical activity again? *Choose the answers that best reflect your situation. Choose only one answer.*

- Yes, for sure
- Probably yes
- I don't know now
- Probably not
- Definitely not
- Don't know / No answer

Q15. If you were to re-start physical activity, why would it be? *Choose the answers that best reflect your situation. Choose only one answer.*

- Be fit
- Exercise is good entertainment for me
- Avoid or manage health conditions
- Improve mood
- Lose or maintain weight
- Improve my body's appearance
- Physical activity lets me have contact with friends and persons I enjoy.
- Improve my self-esteem
- Physical activity gives me a sense of personal accomplishment
- Improve athletic performance
- Participate in social activities
- Sharing activities with other women
- Play with children/grandchildren/nephew/niece
- As a consequence of the confinement during the pandemic
- Exercising increases my acceptance by others
- Gain weight
- Other

Q16. Are you familiar with the *Mugiment* project –which is aimed at achieving an active Basque society, bringing together initiatives to promote physical activity and reduce sedentary lifestyles? *It includes, for example, the Tipi-Tapa programmes, healthy routes, municipal sports guidance services, the use of healthy parks, Mugi Mugi, etc. Choose only one answer.*

- No
- Yes
- Don't know / No answer

Q17. Have you ever taken part in women-only physical activity programmes or courses? *For example, Emakumea Arraunlari, Emakumea Surflari, Emakumea Pilotari, Lilaton, 200Women on bikes, Zarauzko emakume Korrikalariak (ZEK), Azpeitiko emakume Korrikalariak taldea, Segi ezan programme (Hegalak Zabalik Fundazioa), or similar initiatives and groups. Choose only one answer.*

- No
- Yes

If you have participated in a physical activity programme or course intended exclusively for women, please indicate which one.

____

____

DIMENSION 4. FAMILY-LIFE BALANCE FOR LEISURE TIME PHYSICAL ACTIVITY PARTICIPATION

Q18. Are you a mother or do you care for a minor?

- ☐ No (go to question Q20)
- ☐ Yes

Q19. How many children and/or young people do you care for? *How old are they? Please indicate the number for each age group.*

- ☐ 0-2 years: ______
- ☐ 3-6 years: ______
- ☐ 6-9 years: ______
- ☐ 10-12 years: ______
- ☐ 13-17 years: ______
- ☐ 18 years and above: _____

Q20. Do you care for any dependent people? *For example, a relative or elderly person, a person with a disability or a person with an illness (unrelated to your usual profession).*

- ☐ No
- ☐ Yes

Q21. Does your partner help you to be more physically active in your leisure time? *Choose all the answers that best describe your situation.*

- ☐ I don't have a partner
- ☐ Has encouraged me to participate in physical activity
- ☐ Has participated in physical activity with me
- ☐ Has helped me to plan to take part in some physical activity
- ☐ Has taken care of some of my duties so that I can do more physical activity
- ☐ Has taken responsibility for childcare so that I could be more active
- ☐ Has not offered me any help to be able to participate in physical activity
- ☐ Has made it difficult for me to participate in any physical activity
- ☐ Don't know / No answer

Q22. Do your family members and/or friends help you to be more physically active in your leisure time? *Choose as many answers as you want.*

- ☐ Have encouraged me to participate in some physical activity
- ☐ Have participated in physical activity with me
- ☐ Have helped me plan to participate in physical activity
- ☐ Have taken care of some of my duties so that I could be more physically active
- ☐ Have taken responsibility for childcare so that I could be more active
- ☐ Have not offered me any help to be able to participate in physical activity
- ☐ Have made it difficult for me to participate in any physical activity
- ☐ Don't know / No answer
